# Air Pollution and COVID-19: A Possible Dangerous Synergy for Male Fertility

**DOI:** 10.3390/ijerph18136846

**Published:** 2021-06-25

**Authors:** Luigi Montano, Francesco Donato, Pietro Massimiliano Bianco, Gennaro Lettieri, Antonino Guglielmino, Oriana Motta, Ian Marc Bonapace, Marina Piscopo

**Affiliations:** 1 Andrology Unit and Service of Lifestyle Medicine in UroAndrology, Local Health Authority (ASL) Salerno, Coordination Unit of the Network for Environmental and Reproductive Health (EcoFoodFertility Project), “Oliveto Citra Hospital”, 84020 Oliveto Citra, Italy; 2 PhD Program in Evolutionary Biology and Ecology, Department of Biology, University of Rome Tor Vergata, 00133 Rome, Italy; 3 Unit of Hygiene, Epidemiology, and Public Health, Department of Medical and Surgical Specialties Radiological Sciences and Public Health, University of Brescia, 21100 Brescia, Italy; francesco.donato@unibs.it; 4 ISPRA, Italian Institute for Environmental Protection and Research, Via Vitaliano Brancati 60, 00144 Roma, Italy; maxbianco1@libero.it; 5 Department of Biology, University of Naples Federico II, 80126 Napoli, Italy; gennarole@outlook.com; 6 Unit of Reproductive Medicine (UMR), 95030 Catania, Italy; angugl2017@gmail.com; 7 Department of Medicine, Surgery and Dentistry, University of Salerno, 84081 Salerno, Italy; omotta@unisa.it; 8 Department of Biotechnology and Life Sciences, University of Insubria, 21100 Varese, Italy

**Keywords:** COVID-19, SARS-CoV-2, male fertility, semen quality, air pollution, ACE2

## Abstract

Several studies indicate that semen quality has strongly declined in the last decades worldwide. Air pollution represents a significant co-factor with the COVID-19 impact and has negative effects on the male reproductive system, through pro-oxidant, inflammatory and immune-dysregulating mechanisms. It has recently been reported that chronic exposure to PM2.5 causes overexpression of the alveolar ACE2 receptor, the entry route of SARS-CoV-2 into the organism shared by the lungs and testis where expression is highest in the body. In the testis, the ACE2/Ang-(1–7)/MasR pathway plays an important role in the regulation of spermatogenesis and an indirect mechanism of testicular damage could be due to the blockade of the ACE2 receptor by SARS-CoV-2. This prevents the conversion of specific angiotensins, and their excess causes inflammation with the overproduction of cytokines. PM2.5-induced overexpression of the alveolar ACE2 receptor, in turn, could increase local viral load in patients exposed to pollutants, producing ACE2 receptor depletion and compromising host defenses. By presenting an overall view of epidemiological data and molecular mechanisms, this manuscript aims to interpret the possible synergistic effects of both air pollution and COVID-19 on male reproductive function, warning that the spread of SARS-CoV-2 in the fertile years may represent a significant threat to global reproductive health. All of this should be of great concern, especially for men of the age of maximum reproductive capacity, and an important topic of debate for policy makers. Altered environmental conditions, together with the direct and indirect short- and long-term effects of viral infection could cause a worsening of semen quality with important consequences for male fertility, especially in those areas with higher environmental impact.

## 1. Introduction

According to internationally shared knowledge, the outbreak of severe acute respiratory syndrome coronavirus 2 (SARS-CoV-2) infection began in Wuhan, in the Hubei region of China, in December 2019 [1], and has spread all over the world. The World Health Organization (WHO) declared the coronavirus a pandemic on 11 March 2020 [2]. The rapid spread and high infectivity of the virus, indeed, have caused serious problems for the health systems of many countries.

Currently, WHO official estimates (updated to 19 May 2021) are about 165 mln confirmed cases, about 3,42 mln deaths.

As far as the distribution of the contagion and the impact of SARS-CoV-2 concerns, at least four observations are worthy of note:

(A). The virus infects multiple tissues, including the testis [3,4] and a meta-analysis of over three million reported global cases showed that male patients with confirmed Coronavirus disease 2019 (COVID-19) have both a higher requirement for intensive treatment unit (ITU) admission and a higher mortality rate than females [5].
(B). The spread of the Coronavirus (COVID-19) during the first pandemic wave primarily affected the elderly population. Epidemiological data from China, Italy, Japan, Singapore, Canada and South Korea showed that susceptibility to infections in individuals under 20 years of age was about half that of adults over 20 years of age and that clinical symptoms occurred in 21% (95% confidence interval: 12–31%) of infections in people aged 10–19 years, rising to 69% (57–82%) of infections in people older than 70 years [6]. Nevertheless, while the majority of cases in most territories around the world settled into an older age profile, positive cases in Hong Kong were concentrated among younger age groups, with the highest incidence of cases reported in the 15–24 age group [7]. During June−August, the COVID-19 pandemic in the United States affected more young people than during January−May 2020 [8]. The shift toward the younger age group happened in all four U.S. census regions, regardless of changes in incidence during this period, and was evidenced in visits for COVID-19-related diseases, positive SARS-CoV-2 RT-PCR test results and confirmed COVID-19 cases. A similar trend was observed in Europe, where the median age of COVID-19 cases decreased from 54 years in the January−May period to 39 years in the June−July period, during which time persons aged 20 to 29 years accounted for the highest proportion of cases (19.5%) [9]. This shift has also been boosted by the spread of the most significant variants of the coronavirus SARS-CoV-2 from September 2020 to March 2021: the UK, Brazilian, South African, Indian variants. These variants exhibit a greater contagiousness with significant prevalence in young people and children [10].
(C). Several studies point out that chronic exposure to air pollutants in the most polluted areas of the world leads to more severe and lethal forms of COVID-19.
(D). The concept of “mild COVID symptoms”, such as dyspnea, chest heaviness, palpitations and fatigue, muscle pains that reduce people’s ability to perform physical or caring activities, needs to be redefined. Indeed, clinicians do not yet know whether prolonged ill-health following asymptomatic or mild or severe symptomatic infection will generate, long-term, moderate, or heavy effects in the lungs [11] or potentially in other tissues, including the testis.

## 2. Sperm Decline in the World

**Figure 1:**
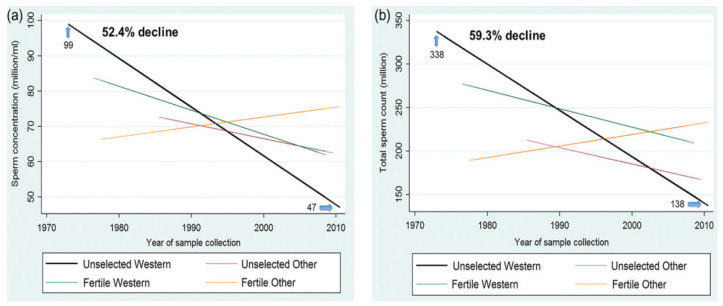
(**a**) Meta-regression model for mean sperm concentration by fertility and geographic groups, adjusted for potential confounders. (**b**) Meta-regression model for mean total sperm count by fertility and geographic groups, adjusted for potential confounders. Reproduced under licence N° 5070710881460 from: Levine H, Jørgensen N, Martino-Andrade A, Mendiola J, Weksler-Derri D, Mindlis I, Pinotti R, Swan SH. Temporal trends in sperm count: a systematic review and meta-regression analysis. Hum Reprod Update. 2017 Nov 1;23(6):646–659, doi:10.1093/humupd/dmx022. PMID: 28981654; PMCID: PMC6455044) [13].

Multiple studies have shown a decreased sperm concentration in many industrialized countries in the last decades, and the incidence of male infertility has increased dramatically (from 7–8% in the 1960s to 20–30% today). In a meta-analysis of 61 European studies in 1992, Carlsen examined semen analyses of donors’ samples from 1934 to 1990 and observed a progressive worsening of the qualitative and quantitative characteristics of semen (from 113 Mil/mL in 1940 to 66 Mil/mL in 1990 and a 19% decline in ejaculate volume) [12]. A systematic review and meta-regression model showed that sperm concentration (SC) and total sperm count (TSC) decreased significantly among men in geographic groups, specifically, in North America, Europe, and Australia, during 1973–2011 [13] (Figure 1).

Specifically, mean SC and TSC decreased by an average of 1.4 and 1.6% per year, with an overall decrease of 52.4 and 59.3%, respectively. In Africa, an overall decrease of 72.58% in sperm concentration was reported from 1980 to 2015 [14]. In Asia, seminal quality in Indian men over the last three decades, has shown a decrease in sperm concentration and normal morphology [15], while for Iranian men, the infertility rate has increased by 20% over the last 20 years [16]. In China, a study carried out between 2001 and 2015 on 30,636 young donors reported a decrease in spermatozoa concentration from 68 × 10^6^/mL to 47 × 10^6^/mL and a reduction of spermatozoa with normal morphology from 31.8 to 10.8% [17]. Another recent study on 9357 healthy young men living in Wuhan reported a relevant decline in sperm count in only 5 years (from 2010 to 2015) [18]. Finally, a median reduction of 0.24 million/mL of spermatozoa per year was reported between 1995 and 2018 in Brazil [19].

## 3. Air Pollution and Impact on Male Fertility

Currently, various environmental factors are negatively associated with male reproductive health, particularly endocrine-disrupting chemicals, which can interfere with normal hormonal balance [20,21,22], and air pollutants [23,24]. For the latter, several epidemiological studies report negative effects on semen parameters [23,24].

A recent experimental animal study (Sprague–Dawley rats) demonstrated that fine particulate matter with a diameter of 2.5 μm or less (PM2.5) alters the integrity of the blood−testis barrier (BTB) through excessive ROS-mediated autophagy that destroys tight junctions, adherens junctions and gap junctions [25]. Regarding the negative impact of individual pollutants on spermatogenesis, it has been observed that chronic exposure to nitrogen dioxide (NO_2_) and sulfide dioxide (SO_2_) levels is negatively associated with sperm count, motility, and even testicular volume in infertile subjects. In addition, it has also been reported that spermatogenesis improves [26] with reduced levels of SO_2_. Using linear multivariate distributed lag models to identify windows of exposure to air pollution susceptibility during the spermatogenic cycle, a Chinese pre-proof study recently found in male residents of Wuhan that exposure to particulate matter with a diameter of 10 μm or less (PM10) during spermatidogenesis (from 6 at 12 weeks) was associated with reduced sperm concentration and total and progressive motility of spermatozoa [27]. In contrast, exposure to SO_2_ during the period of spermatocytogenesis (0 to 5 weeks), was negatively associated with sperm concentration and total spermatozoa motility, while exposure to ozone (O_3_) during the spermiogenesis period (11 to 12 weeks) was associated with low sperm count. All of these findings confirmed that air pollution impairs all phases of the spermatogenetic cycle through various pathways [28], and these more specific studies on the impact of individual air pollutants on spermatogenesis confirm the epidemiological evidence that air pollution may act on one or more seminal parameters [29].

Several studies report the relationship between exposure to air pollutants and genetic damage to male germ cells [29,30] and epidemiological studies indicate that the quality of human semen may reflect the health status of men [31,32,33,34]. The first systematic study conducted in the Czech Republic within the research program “Teplice”, found increased DNA fragmentation and increased rates of sperm aneuploidies. Both processes were associated with high concentration of polycyclic aromatic hydrocarbons (PAHs) and PM10 in the air and the recurrent identification of air pollutants in the semen [35]. The authors hypothesized that DNA adducts, produced by PAHs, might be present in the semen of individuals exposed to heavy pollution, causing increased fragmentation of sperm DNA [36]. In the same research program, Rubes and colleagues confirmed the involvement of air pollutants in sperm DNA damage in young males from Teplice after periods of exposure to both low and high intermittent air pollution [37]. In this regard, we have recently reported, through molecular investigations, that the sperm nuclear basic proteins from samples of men living in polluted areas have a novel and unexpected behaviour, being involved in oxidative DNA damage [38].

The potential mechanisms through which air pollutants may exert their effects on male reproduction is through pollutants that act as endocrine disruptors or that induce genetic and epigenetic changes and oxidative stress [24].

Regarding the mechanism of endocrine disruptors, chronic exposure to PM2.5 in animal models has demonstrated a significant decrease in follicle-stimulating hormone (FSH), low circulating testosterone levels and consequent reduction in sperm concentration through suppression of the hypothalamus−pituitary−gonad axis (HPG) [39,40].

Epigenetic effects on male gametes by particulate matter have been demonstrated in animal studies; in particular those concerning DNA methylation in mice sperm [41]. Heavy metals can be accumulated in human semen and their associations with sperm quality parameters, particularly motility, morphology, integrity of DNA strand, telomere length and DNA methylation, have been reported [42,43,44,45].

However, oxidative stress, due to an excess of reactive oxygen species (ROS), unbalanced by the presence of reductive activity, may be the common denominator through which air pollutants can result in biomolecular-level damage to DNA, proteins, lipids, leading to chronic low-grade inflammation [24,46]. Spermatozoa are extremely sensitive to the pro-oxidant action of contaminants due to the richness of polyunsaturated fatty acids in their plasma membrane, known to be targets of ROS, and the low intracytoplasmic presence of antioxidant enzymes due to the small cytoplasmic volume [41].

Considering the role of angiotensin-converting enzyme 2 (ACE2) in the male reproductive system and the relevance of this enzyme in containing the inflammatory response in the lungs as a consequence of particulate matter 2.5 (PM2.5) exposure [47], it would be important to investigate whether the particulate matter would be able to generate injuries in the testis or alterations in the sperm physiology, activity morphology in ACE2 knockout mice, thus suggesting a protective function of ACE2 also for this anatomic compartment.

## 4. SARS-CoV-2 Impact on the Male Reproductive System

People of all ages are generally susceptible to COVID-19, showing a high prevalence in asymptomatic individuals. The most vulnerable groups to complications are the elderly people, especially those with other co-morbidities, subjects with chronic diseases or low immune defences, pregnant women and new-borns [48,49].

The main concern about the possible impact of SARS-CoV-2 on the male reproductive system is based on the entry route of the virus into the organism. Like other viruses (papilloma, HIV, hepatitis), SARS-CoV-2 could bypass the blood−testes barrier and cause testes damage, up to infertility [50,51]. The virus exploits the ACE2 receptor found in different tissues and causes the multiorgan impact of COVID-19. The ACE2 receptor is also found in the testis and, according to the Human Protein Atlas portal (https://www.proteinatlas.org), the expression level in this tissue is among the highest in the human body, while TMPRSS2, the type II transmembrane serine protease required to cleave ACE2 and process the viral S protein thereby enhancing virus entry [52] has been shown to be expressed in many male tissues, such as the ductus deferens, epididymis, seminal vesicle, and prostate [53]. In addition, according to a study of scRNA dataset of three adult human testis samples, TMPRSS2 expression was concentrated in spermatogonia and spermatids and expressed at relatively low levels in other cells [53]. In addition, a recent study has shown that ACE2 and TMPRSS2 co-express in prostatic hillock and club cells. Other authors also discovered TMPRSS2 in spermatogonia and spermatids, as well as ACE2 in Leydig and Sertoli cells and spermatogonia. This discovery sheds some light on SARS- CoV-2 sensitivity to male reproductive system infection [54]. Chen and colleagues have shown that the protein is released into semen in proteasomes [55]. However, results from many laboratories have failed to identify the co-expression of the TMPRSS2 modulatory protein in testicular cells, sperm or oocytes, suggesting that gametes are unable to transmit SARS-CoV-2 [56]. These results suggest that the virus may infect testicular cells, although further evidence is needed, while not excluding the possibility that overall, the virus may bind to the highly expressed ACE2 protein. The possible presence of SARS-CoV-2 virus in the seminal fluid has very recently been reviewed by Borges et al. [57] and the results are controversial.

**Figure 2:**
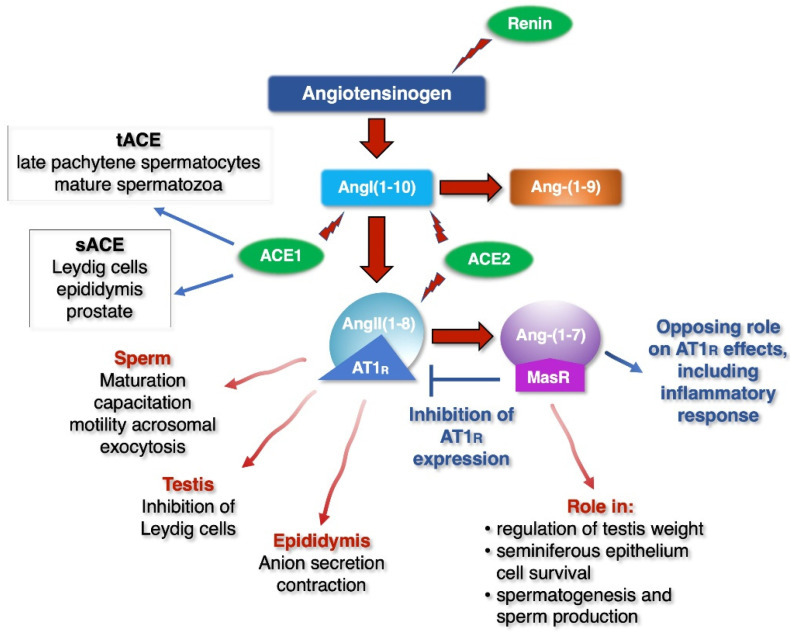
Simplified view of the renin–angiotensin system (RAS) in the male reproductive system. Schematic cartoon illustrating the renin–angiotensin system in the male reproductive system. The main components of the pathway and the main functions of the ligands and receptors are shown. Renin enzyme cleaves its substrate Angiotensinogen to form the decapeptide AngI, which is in turn cleaved by ACEI to produce AngII(1−8). The functions of AngII(1−8) in male reproductive events are stimulated by the AngII type 1 receptor (AT1R). For the details, see the text. Full red arrows indicate the enzymatic action on the substrate. The blue block arrow indicates the inhibition of MasR on AT1R.

**Figure 3:**
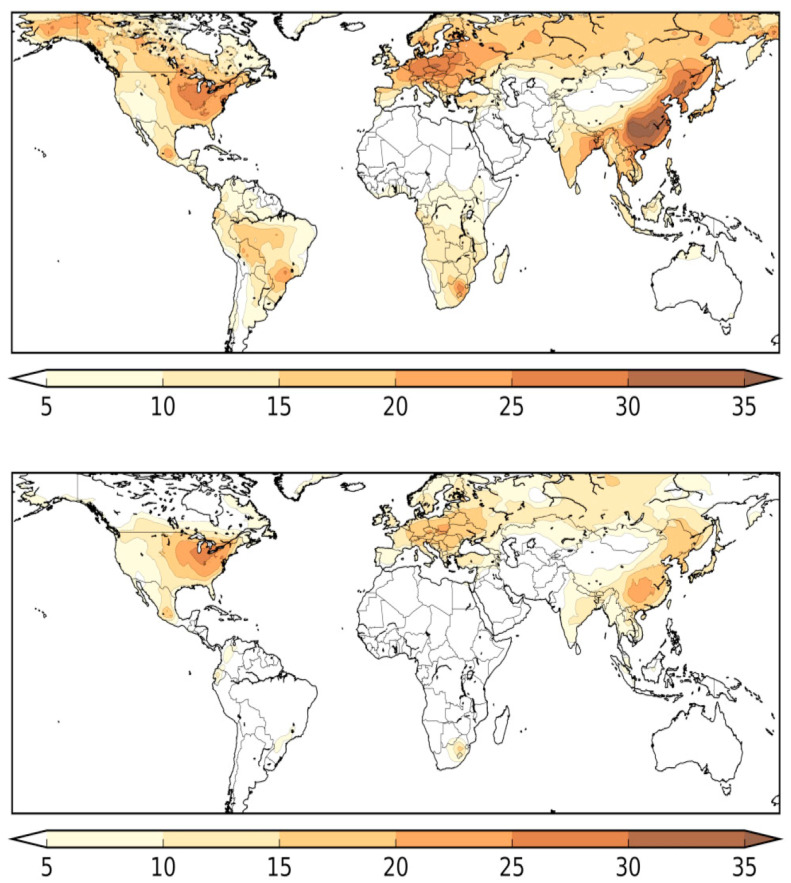
Estimated percentages of COVID-19 mortality attributed to air pollution from all anthropogenic sources (**top**), and from fossil fuel use only (**bottom**). The regions with high attributable fractions coincide with high levels of air pollution. The mapped results account for population density, thus reflecting population weighted exposure to PM2.5. Reproduced under licence N° 5070720206174 from: Pozzer A., Dominici F., Haines A., Witt C., Münzel T., and Lelieveld J. Regional and global contributions of air pollution to risk of death from COVID-19. Cardiovascular Research (2020) 116, 2247–2253, doi:10.1093/cvr/cvaa288 [116].

A pathophysiological impact on the testes following SARS-CoV-2 infection has been suggested: the testosterone-to-LH ratio seems to be dramatically reduced, indicating that SARS-CoV-2 may have an impact on Leydig cells, which infer to the potential hypogonadism [58]. A classical, locally generated and complete renin–angiotensin system (RAS) is operative in the testes, where angiotensinogen, the precursor of angiotensins, has been identified (reviewed in [59]) and two different angiotensin-converting enzymes (ACE) generating AngII are active: the somatic form (sACE) is present in the Leydig cells, epididymis and prostate (Figure 2) [59], while a specific testis variant (tACE or ACET) is exclusively found in late pachytene spermatocytes and mature spermatozoa [59]. AngII functions in male reproductive events are stimulated by the AngII type 1 receptor (AT1R) (Figure 3). This receptor mediates several important fertility functions, ranging from anion secretion and contraction in the epididymis, maturation, capacitation, acrosomal exocytosis and motility in sperm, inhibition of Leydig cells in the testis [59]. Studies on ACE enzyme knock-out animals showed that only *tAce1^−^/^−^* male mice are sterile, whereas *sAce^−^/^−^* animals were fertile [60].

The ACE2 enzyme then converts AngII into the metabolite Ang-(1–7), which acts as a ligand for the Mas receptor (MasR). The MAS receptor elicits signalling opposing Ang II/AT1R effects, including the inflammatory ones, and inhibiting AT1R expression [61]. Both ACE2 and MasR are present in human Leydig and Sertoli cells (Figure 2). The ACE2/Ang-(1–7)/MasR pathway regulates several important functions in the male reproductive system, including steroidogenesis, spermatogenesis and erection, thus consistently contributing to the male reproductive system [59]. ACE2 (*Ace2^−^/^−^*) and MasR (*Mas^−^/^−^*) knock-out mice are both fertile, but testis weight in *Mas^−^/^−^* animals are reduced and the seminiferous epithelium shows a greater volume of apoptotic cells, giant cells and vacuoles. In *Mas^−^/^−^* mice, apoptosis increases during meiosis and Sertoli cell efficiency, the meiotic index, overall spermatogenesis rate and pro die sperm production are all lower, suggesting an important role of the ACE2/Ang-(1–7)/MasR pathway in the regulation of spermatogenesis [62]. Using an Ang-(1–7) antagonist (A-779), Leal and colleagues further confirmed these results, since the chronic infusion of the compound increased the number of apoptotic cells and primary spermatocytes [62]. Moreover, studies on infertile men with nonobstructive azoospermia and impaired spermatogenesis, have demonstrated the absence of both Ang-(1-7) and MasR in the seminiferous tubules. Comparing infertile men with nonobstructive or obstructive azoospermia, the former expressed lower levels of the MAS and ACE2 messengers [63]. Very recently, Valdivia and colleagues have suggested that the ACE2/Ang-(1–7)/Mas axis is localized in the acrosome region, as well as in the spermatozoa tail. This axis activates the PI3K/AKT pathway in a dose-dependent manner and improves the motility of asthenozoospermic patients, without modulating the acrosome reaction [64]. Finally, the signalling through MasR opposes AngII/AT1R effects, also by inhibiting AT1R expression.

It is important to point out that SARS-CoV and MERS-CoV share the same ACE2 receptor with SARS-CoV-2. SARS-CoV and MERS-CoV, however, were less infectious than SARS-CoV-2, in fact SARS-CoV-2 binds ACE2 with a 10- to 20-fold higher affinity than other SARS coronaviruses [65], and SARS-Cov and MERS-CoV had limited epidemic impact with only 8098 and 2538 people, respectively, infected globally (WHO website), while the number of confirmed cases of SARS-CoV-2 infection is over 126 million as of 26 March 2021. Considering the common viral mechanisms of immunological damage, redox imbalance and associated inflammatory processes, it is worth noting that significant reproductive damage was observed in some men during the SARS outbreak in 2002 and 2003 [66]. The histological picture showed the diffuse destruction of the germ epithelium, a rare number of spermatozoa in the seminiferous tubules and a significant increase in the interstitial tissue of CD3+ lymphocyte T and CD68 macrophages with abundant precipitation of IgG in the seminiferous epithelium. According to immunohistochemical evaluation, all signs of immune-mediated orchitis were present [66]. Testicular impairment has also been reported from other infections with pulmonary tropism in roosters after infectious bronchitis virus (IBV) and avian metapneumovirus (aMPV), thus reducing the fertility of chickens [67]. In China, many concerns have been reported about certain aspects of the male reproductive system in individuals cured of COVID-19. Some authors investigated pathological changes and whether SARS-CoV-2 could be detected in the testes of patients who had died from COVID-19. They found that the testes from COVID-19 patients exhibited significant seminiferous tubular damage, reduced Leydig cells and mild lymphocytic inflammation [68]. These findings were confirmed by other studies in which the autopsied testicular and epididymal samples of COVID-19 revealed the presence of interstitial edema, congestion, and exudation of red blood cell in the testes and epididymides. Thinning of the seminiferous tubule was also reported. In addition, the apoptotic cell number within seminiferous tubules was markedly higher in COVID- 19 than in control cases [69]. In line with these observations, other authors have found prominent leukocyte infiltration, including CD3^+^ T lymphocytes, CD20^+^ B lymphocytes, CD68+ macrophages, HLA-DR+ myeloid cells, and CD38^+^ plasma cells in the testes of COVID-19 patients [70]. All this evidence of localized testicular damage suggests the potential for adverse reproductive consequences at the anatomical, cellular, and molecular levels [71].

The higher prevalence, severity and duration of infection in males compared to females could be attributed to the greater presence of ACE2 receptors in the testis compared to the ovary, particularly in spermatogonia, Leydig and Sertoli cells, which some authors have considered testicular viral reservoirs [72], although there is no conclusive evidence for the presence of the virus in the testes. These findings, together with the increased contagiousness of SARS-CoV-2, may suggest that the virus is capable of causing testicular damage [63,73,74,75]. The blood−testis barrier (BTB) plays an important role in protecting the testicular cells from germ cells and inflammation. The BTB is also necessary for Sertoli cells to control the adluminal environment. During the acute phases of COVID-19, the BTS cannot protect the testicular cells. This causes cytokine activation, resulting in a cytokine storm that leads to the destruction of the tubule epithelium in the seminaries. SARS-CoV induces severe orchitis by producing widespread IgG precipitation in testicular tissue, resulting in testicular leukocyte infiltration and germ cell destruction from the standpoint of antibody generation [76]. To date, and to the best of our knowledge, few studies have been conducted to assess the impact of SARS-CoV-2 on the male reproductive system. In addition to the prolonged fever which can per se temporarily reduce male fertility [77], possible direct damage caused by the virus is supported by excessive systemic cytokines. Therefore, indirect and substantial immune damage due to increased levels of ACE2, which have a proinflammatory action, has been hypothesized. This is because the ACE2 receptor, blocked by the virus, prevents the conversion of specific angiotensins, and the excess of these causes inflammation with excessive cytokine production [78,79]. One of these, IL-6, induced by Sars-CoV-2, can cause leukocyte infiltration into the testis with disruption of the testis−blood barrier that can lead to an autoimmune response with potential formation of antisperm antibodies (ASA) [80,81]. Moreover, inflammation itself, by inducing excessive production of radical oxygen species (ROS), can lead to sperm DNA damage. Indeed, the harmful effects of ROS on semen quality are widely documented and also involve peroxidation of sperm membrane lipids and the induction of apoptotic pathways in spermatozoa [82,83]. The recent hypothesis that SARS-CoV-2 itself or via ACE 2 may directly interfere with the autophagy pathway to achieve virus survival is suggested by the increased level of autophagy receptor SQSTM1/p62 in SARS-CoV-2-infected cells, and this could represent another possible mechanism of damage to male reproductive function [84]. In addition, a recent retrospective study of males affected by COVID-19, found significantly high levels of LH and prolactin that could have resulted from an initial reduction in testosterone production that then increased LH levels due to negative feedback [85]. This hormonal imbalance could depend on the impact of the virus, both at the direct testicular level, at the level of Leydig cells and also on the hypothalamus−pituitary−gonad (HPG) axis. Neuro-invasion by SARS-CoV-2 has been demonstrated by autopsy examination of deceased patients with marked neurological symptoms. Neurons and glial cells express ACE2 and neuroinflammation may involve several brain regions including the hypothalamus that controls the production of GnRH and consequently the secretion of LH and FSH) from the anterior pituitary. The high levels of LH and FSH in COVID-19 patients could indicate an activation of the gonadotropin-producing cells due to surplus levels of inflammatory cytokines [86,87].

If persistent, this condition may lead to clinical hypogonadism or earlier to spermatogenetic alteration.

The reproductive health of patients with chronic viral infections can be seriously compromised. Several viruses such as influenza, Ebola, Zika, Epstein−Barr have been found in human semen and can penetrate spermatozoa. Some of these viruses can also be transmitted vertically to offspring via the male germ line, and can impair sperm function in the next generation [88]. Hepatitis B virus (HBV), for example, has been found to adversely affect human sperm function, and the asialoglycoprotein receptor (ASGP-R) and HBV S protein (HBs) were found to be mainly localized in the postacrosomal region. In particular, ASGP-R may play a role in HBs uptake into sperm cells. The infection of germ cells by HIV at the beginning of spermatogenesis has been reported, resulting in clonal transmission of the virus into spermatozoa, as sperm bind to somatic cells via HLA-DR [89,90]. Researchers from the Perelman School of Medicine at the University of Pennsylvania [91] reported that amyloid fibrils in human semen significantly increase Ebola virus infection and protect the virus from harsh environmental conditions, such as heat and dehydration.

Only three contradicting studies, however, have investigated the presence of SARS-CoV-2 in this biological fluid [92]. The first, conducted on 34 adult male patients one month after SARS-CoV-2 infection, reported the absence of the virus in human semen [93]. A second, designed as a pilot cohort study, examined 8 semen samples from adult men who recovered between 8–54 days, symptom-free, 2 from men in an active phase of COVID-19 infection, and 14 healthy volunteers negative for SARS-COV-2 infection as a control group. No SARS-CoV-2 RNA was detected in the semen samples of the viral RNA positive subjects in the pharyngeal swab [94]. In contrast, a third study detected the virus in semen in 15.8% of 38 adult Chinese males, including those convalescing from COVID-19 [95]. The authors, however, were unable to determine the survival time of the virus and its shedding, and the viral concentration in semen.

In the absence of convincing evidence of the presence of SARS-CoV-2 in the sperm, its possible transmission and the direct and indirect consequences of the infection, COVID-19-positive men are strongly advised to wait three months before starting any ART procedure or performing clinical tests, spermiograms or hormone evaluations [96]. These data underscore the importance of definitively assessing whether SARS-CoV-2 is present in the testes and semen and what impact COVID-19 might have on male reproductive function. It is extremely important to evaluate these effects, especially in reproductive age, taking into account that young patients have higher expression of the ACE2 receptor than older patients [97], making them more vulnerable to gonadal involvement by SARS-CoV-2, and that the mean age of infection has progressively lowered, especially with the increased prevalence of virus variants that are affecting more people of fertile age [98]. Moreover, if few studies, mainly on the acute phase, report fertility damage in males with COVID-19, the direct and indirect effects of SARS-CoV-2, considering the points mentioned above, suggest the possibility of long-term effects on male reproductive health. All this, is of a great concern for the possible consequences on the reproductive future of new generations affected by COVID-19, also for the immense and not yet estimated impact of the COVID-19 pandemic on several countries around the world.

## 5. SARS-CoV-2 and Pollution

Currently, several studies suggest that air pollution may represent a significant co-factor in determining the rate of spread and mortality in the outbreaks of COVID-19 [99], especially in those cities with high levels of air pollutants where other unfavourable conditions such as higher temperature and humidity, greater population density [100] and commercial exchanges [101] (both accounting for human-to-human transmission mechanisms) contribute significantly to the outbreak of virus. The hypothesis is that air pollutants and particular climatic conditions, especially indoors, may promote longer residence of viruses in the air. This could promote an “indirect” as well direct (individual to individual) spread and this observation has been suggested for both SARS-CoV-1, during the 2003 infection [102], and SARS-CoV-2 [100].

Several papers have suggested that the interaction of particulate matter (PM) with the virus may pose a potential risk of virus spread, although this has not yet been fully demonstrated [103,104,105]. Industrial regions with the most severe pollutant levels have significantly high mortality rates for SARS-CoV-2 transmission [106,107,108,109]. In Europe, a spatial analysis study of 66 administrative regions in Spain, Italy, France and Germany reported a 78% CFR (3487 on 4443 fatality cases). In central Spain and five regions of the Po Valley in Northern Italy, which have very high levels of particulate matter with a diameter of 10 μm or less (PM10), a diameter of 2.5 μm or less (PM2.5) and nitric dioxide (NO_2_), also have the highest case fatality rate (CFR) of COVID-19 in the western countries [110,111,112]. An association between air pollution indexes and SARS case mortalities has already been described in China during the SARS infection in 2003, with twice the mortality rate in the most polluted areas than in the least polluted areas [113]. Recently, a higher CFR of COVID-19 was reported in Wuhan, China, with increasing concentrations of air particulate matter (PM10 and PM2.5) on a time scale, after adjusting for relative humidity and temperature values [114]. A very recent study of 615 cities on 6 continents, while pointing out that factors such as population size, elderly population, extreme poverty and income level can influence the spread of COVID-19, also showed that PM2.5, NO_2_ and O_3_ under particular meteorological conditions, such as dew, wind gust, pressure and wind speed increase the spread of COVID-19, promoting its adverse health effects and subsequently inducing mortality rates [115].

Excess of mortality from cardiovascular and pulmonary diseases is a strikingly common feature of COVID-19 and air pollution. By characterizing global fine particulate exposure, based on satellite data and calculating the anthropogenic fraction, Pozzer and co-workers [116] estimated the mortality from COVID-19 that could be ascribable to long-term exposure to fine particulate air pollution. They found that anthropogenic air pollution contributed to ~15% of global mortality due to COVID-19, ~27% in East Asia, ~19% in Europe, and ~17% in North America. Most interestingly, in Australia, where air quality regulations do not allow annual PM2.5 limits above 8 mg/m^3^, mortality from COVID-19 attributable to all anthropogenic emissions was found to be ~3%. Figure 3 illustrates how the regions of the world with a high mortality fraction coincide with the weighted population exposure to PM2.5.

After all, the impact of climatic factors (including temperature, precipitation and humidity) on viral circulation and in particular for respiratory syncytial virus (RSV) bronchiolitis have been statistically analysed for some time to help determine the optimal timing of appropriate strategies to prevent RSV outbreaks, taking into account the specific climatic parameters of a given area [117].

The ACE2 receptor has been shown to play a role in recovery from acute lung injury (ALI) generated by the instillation of fine particulate matter with a diameter of 2.5 μm or less (PM2.5) into the lungs of wild type and ACE2 knockout mice. ACE2 expression levels increased in PM2.5-exposed wild type animals and the resting respiratory rate (RRR) and IL-6, TGF-β1, TNF-α and MMP9 levels in ACE2 knockout animals were higher than wild type animals five days after the last administration of PM2.5, suggesting that ACE2 might have a protective role against the PM2.5-induced inflammatory process [47]. Sequestration of ACE2, accumulation of AngII(1-8) with subsequent enhancement of the AT1R receptor, of Ang-(1−7) levels could therefore represent additional mechanisms for the inflammatory consequences induced by SARS-CoV-2 infection.

## 6. Epidemiology and Mechanisms of Damage by Air Pollutants

Currently, several epidemiological studies indicate that air pollution is among the major risk factors for cardiovascular and chronic degenerative disease, premature deaths and reproduction dysfunctions [118] along with lifestyle, as reported in the European Code against cancer [119]. In this regard, a recent review in the rodent model described the mechanisms of cardiovascular diseases, pulmonary oxidative stress and particulate-related inflammation [120]. In particular, chronic exposure to the finest fraction of particulate matter (PM2.5 or less), not only induces inflammation in the alveolar district, but, passing the alveolar barrier, reaches the blood and then the peripheral tissues, inducing oxidative stress both directly and through the excessive production of reactive oxygen species (ROS). This contributes to the activation of inflammasome and, particularly, NLRP3, which influences the maturation and secretion of cytokines, such as IL-1 beta and IL-18, both of which are involved in the systemic inflammatory syndrome and all conditions that facilitate pathogen virulence, including vascular discharge and coagulopathy [121]. It is therefore plausible that inflammatory mediators produced in the lungs by air pollutants may have a systemic impact and facilitate silent systemic inflammation. As a result of exposure to airborne particulate matter, in fact, signs of systemic oxidative stress have been found [122]. How pollutants induce oxidative stress, making the organism more susceptible to complications from pathogens including SARS-CoV-2, is a topic of great interest. Susceptibility to certain pollutants has been shown to increase from one generation to the next in mice [123], and early results appear to indicate the transgenerational effects of pollutants on molecular alterations in the sperm level of men living in polluted areas [124]. The presence in the current generation of damage that occurred in the previous generation suggests that these latter processes may also have a transgenerational basis. Indeed, a particular concern is the increasing susceptibility to noncommunicable diseases (NCDs) and the significant decline in body defences. Taken together, this seems to give a new interpretation to the exponential burden of disease worldwide, which could be interpreted not only from a chronic-degenerative perspective but also as a consequence of the involvement of infectious agents (dengue, yellow fever, etc.) [123,125,126,127]. Experimental evidence has shown, in fact, that dioxins and other pollutants can alter the immune response of the host, which in turn can be transmitted to subsequent generations, reducing the host’s ability to defend against viral pathogens [125].

## 7. Mechanism of Damage of SARS-CoV-2

A process of immune dysregulation with excessive cytokines, typical of the cytokine storm, was the pathogenetic factor of extrapulmonary complications during the 2009 influenza A (H1N1) and avian (H5M1) [128]. In fact, high levels of TNFa, interleukin-6, and interleukin-8 were observed in the lungs, brain, spleen, pancreas, heart, jejunum, and liver [129]. Even if the infectious virus could enter the central nervous system (CNS) and cause damage [130], this would be mediated by an immune-mediated response [131]. For example, TNFa is known to increase blood−brain barrier permeability and induce cell death [132] and IL-6 production in the CNS has been associated with convulsions [133].

Very recently, it has been shown that treatment with myo-inositol may be effective in reducing the IL-6-dependent inflammatory response and improve oxygenation in patients with severe ARDS from SARSCoV-2. Furthermore, the action of myo-inositol on IRE1 endonuclease activity also may inhibit SARS-CoV-2 replication, as has been reported for respiratory syncytial virus [134]. In addition, it has been proposed that monoclonal antibodies that target IL-6 or drugs that can downregulate IL-6 may be effective in blocking inflammatory storms, thus representing a potential treatment for severe COVID 19 patients [135].

Overall, these data show that extra-respiratory tissues actively contribute to the proinflammatory cytokine response during severe infections, and this could contribute to worsening the patient’s clinical picture, with even lethal consequences. SARSCoV-2, similar to other retroviruses (e.g., HIV1) can integrate into the genome [136] and could generate as yet unknown long-term effects.

These viruses, including SARS-CoV-2, have been shown to be able to trigger a rapid process of autoimmune dysregulation, inducing a significant cytokine synthesis, mainly TNF-α, IL-6 e IL-1β, IL-17, IL-18, in genetically predisposed individuals [137]. This process might be even more relevant when exposure to environmental factors alters the regulatory mechanisms for controlling cytokine synthesis and release, leading to their dysregulation and overproduction with over-response of innate and adaptive mechanisms [138,139]. In addition, cytokine release and/or the presence of certain IL-6 polymorphisms may make individuals more susceptible to viral complications, as in specific populations or ethnic groups [140].

Interestingly, in patients with juvenile systemic idiopathic arthritis (SJIA), pulmonary involvement triggers a hyperinflammatory reaction and the appearance of secondary hemophagocytic lymphohistiocytosis (sHLH), as IFN, IL-1β, and IL-6 signature up-regulation can be observed [141,142]. Of note, Mehta et al. suggested that the severity of COVID-19 is associated with a cytokine storm syndrome similar to sHLH [143]. For sHLH, it has been hypothesized that environmental factors may also trigger or exacerbate an aberrant innate and acquired immune response with massive cytokine synthesis in genetically susceptible individuals [144]. Alteration of the ACE2 enzyme activity has been indicated as an important trigger of the pathologies seen in COVID-19 patients [61]. Inactivation of the ACE2 complex leads to thrombosis in infected and noninfected tissues, aberrant sensory and neurological perception in COVID-19 patients and, most importantly, to necrosis in pulmonary, endothelial, and cardiac cells.

Indeed, the severe pulmonary and systemic clinical picture indicates a hyperinflammatory syndrome, called hemophagocytic lymphohistiocytosis with a cytokine storm characterized by increased levels of IL-2, IL-7, granulocyte-inducing factors (granulocyte colony stimulating factor), interferon-γ, inducible protein 10, monocyte chemoattractant protein 1, macrophage inflammatory protein 1-α, e TNF-a6 (tumour necrosis factor-α.6) [143]. Recently, viral infection of endothelial cells leading to endothelitis with a major inflammatory response and systemic impairment of microcirculatory function has also been reported. These pathogenetic mechanisms lead to multiple organ dysfunction [145,146].

## 8. Male Fertility: The Possible Dangerous Synergy of Air Pollution and COVID-19

Although environmental pollution is ubiquitous, there are areas of the world where greater environmental pressure corresponds to a higher incidence of both chronic degenerative diseases and infertility. Moreover, this correspondence is also found between areas of the same country or even the same county/region [147,148].

Ubiquitous exposure to chemicals has increased in the general population of France since the 1950s [149]. In the biological matrices of the French, all major endocrine disrupting chemicals (EDC) have been detected and some of them (non-dioxin-like PCBs, pesticides and triclosan) were found in higher concentrations than in subjects from other countries [150]. A very similar situation has also been observed in the “Land of Fires”, in the Campania Region in Italy, where, in recent decades, the growing incidence of male reproductive system disorders has focused the attention on possible environmental risk factors [42]. These observations may be consistent with the hypothesis recently established in an international report that points to endocrine disruptors as an important cause of reduced semen quality [151]. The worldwide decrease in sperm concentration and morphology, therefore, could to be due, at least in part, to changes in the environmental exposure of men living in highly polluted areas and to a possible transgenerational effect [151].

Spermatozoa are the first to suffer from environmental insults and can be considered early and sensitive markers of environmental exposures [42,44,148] for human [97,132,152], and marine organisms in high environmental impact areas [153,154,155,156]. Since human semen is an early and sensitive health marker [112], spermatozoa may be more susceptible to the impact of SARS-CoV-2, which might further increase reproductive risk in the infected male population [157,158].

In addition to the assessment of virulence and severity of viral impact, reproductive biomarkers, particularly seminal biomarkers, could therefore represent good biological indicators for the assessment of environmental damage to human health, including male fertility [157,158].

Although medium- and long-term clinical information on the current virus impact on the male reproductive system is still limited, this aspect cannot be neglected, particularly in subjects at the age of their highest reproductive activity (of their peak reproductive activity), around 30 years of age, when the expression levels of ACE2 receptors are higher than in subjects aged 20 or 60 [95]. In fact, the renin-angiotensin system is also present in the testes and epididymis and, if altered, leads to the failure of spermatogenesis [63].

Some authors have attempted to hypothesize the mechanisms by which environmental pollution might increase COVID-19 symptomatology [159], suggesting a “double-hit hypothesis”: chronic exposure to PM2.5 causes alveolar ACE2 receptor overexpression, which in turn may increase local viral load in pollutant-exposed patients, producing ACE2 receptor depletion and compromising host defences. High levels of atmospheric NO_2_ may provide a second hit, causing a severe form of COVID-19 in ACE-2-depleted lungs with a subsequent worsening of outcome [160].

**Figure 4:**
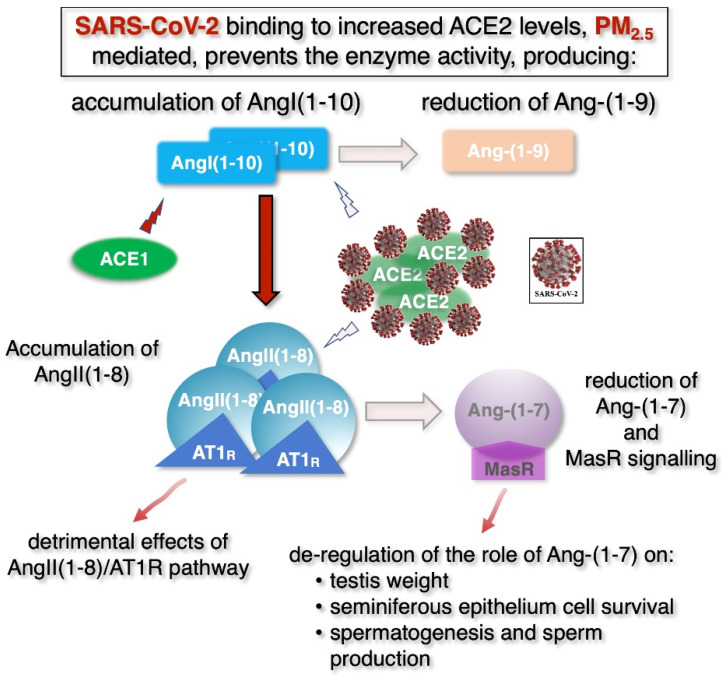
Speculative model of synergistic action of PM2.5 and SARS-CoV-2 on the renin-angiotensin pathway in male reproductive system. In polluted areas, high levels of PM2.5 may produce harmful synergistic effects with a SARS-CoV-2 infection. See text for details. Lighter colors indicate reduced amounts of the indicated molecules and extent of the enzymatic reaction (arrows), respectively.

In theory, this evidence could also be applied to the male reproductive system, speculatively suggesting that the impact of COVID-19 on male fertility could be greater in polluted areas, where pollutant levels may promote the severity of SARS-CoV-2 infection [92] and explain the synergistic adverse effects on male fertility due to the simultaneous presence of air pollution and the virus. In the testis, high local physiological levels of the ACE2 enzyme, which have been reported to grow in the testicles of infertile men [161], could play a protective role, by acting in two ways (Figure 4): (i) by lowering AngII(1−8) levels and reducing its signalling through the AT1 receptor and (ii) by generating Ang-(1−7), thus activating the Mas receptor-dependent pathway and limiting the damaging effects of precursor accumulation AngI and AngII. In polluted areas, high levels of PM2.5 could produce harmful synergistic effects with SARS-CoV-2 infection. Together with the previously mentioned excessive ROS-mediated autophagy [25], PM2.5 might increase the already high levels of ACE2, which, in turn, might be sequestrated by the virus which would inactivate the enzyme. Higher ACE2 levels might be sequestrated by the virus which would inactivate the enzyme. This would produce several effects: (1) reduced production of Ang(1−9) and consequent accumulation of AngI(1−10); (2) accumulation of AngII(1−8); (3) overactivation of the ACE/AngI-AngII/AT1R pathway; (4) strong reduction of the ACE2/Ang-(1-7)/MasR pathway. Together, these mechanisms would cause a strong inflammatory response and impair the physiological effects of the MasR signaling on spermatogenesis (Figure 4).

To confirm this hypothesis, however, further research is needed to definitively demonstrate the presence of the virus in the male reproductive system and its persistence after the acute infection phase. Other aspects could impact on fertility. Pollutants share common intracellular targets with the RNA viruses, represented primarily by mitochondria, which are very abundant in spermatozoa. Mitochondrial DNA is very sensitive to the effects of pollutants, and SARS-CoV-2 has been shown to interact in its very early stages of intracellular invasion with some mitochondrial proteins whose downregulation seems to determine, through a complex system of mutual interactions, yet to be clarified, an energy dysfunction with alterations in the oxide/reductive balance, i.e., the triggering of an excessive production of reactive oxygen species (ROS), an inflammatory process, an alteration of apoptosis and immunoregulatory mechanisms [162,163].

## 9. Conclusions

In conclusion, attention to reproductive aspects should be a major concern for policy makers in this pandemic, as the dramatic decline in male fertility worldwide, along with high levels of pollution, create risky conditions that could further compromise the reproductive capacity of the human species.

COVID-19 with its enormous prevalence in the world population could represent a strong accelerator of male fertility decline, mainly because of the progressively lowering of the average age of infection and because COVID-19 increases oxidative stress, which is a major cause of infertility.

Moreover, new variants of Sars-CoV-2, such as the English, South African, Brazilian and Indian ones, are an additional concern because they make the virus more infectious and widespread starting with young people, creating conditions for greater disease.

Therefore, in areas where the pollution rates are higher, more attention should be paid to fertility health, considering that spermatozoa are the first sentinels of environmental health and may be more susceptible to the potential synergistic action of pollution and SARS-CoV-2 infection.

Therefore, it is essential to undertake extensive research to reveal the exact impact and mechanism by which the pandemic COVID-19 might affect male fertility parameters, it is also necessary to investigate the possible synergy between COVID-19 and air pollution on male fertility, including young men and those who already have difficulty conceiving by means of assisted reproduction technology, focusing on the molecular aspects of infertility in addition to the classic seminal reproductive parameters, since in several cases the classical spermiogram parameters may not be sufficient to reflect a man’s fertility status. Given that testicular cells play critical roles in the transmission of genetic information between generations, there is growing concern about the effects of COVID-19 on testicles, making it essential to investigate the transgenerational effects of pollutants on susceptibility to COVID-19.
